# YjbH Requires Its Thioredoxin Active Motif for the Nitrosative Stress Response, Cell-to-Cell Spread, and Protein-Protein Interactions in Listeria monocytogenes

**DOI:** 10.1128/JB.00099-20

**Published:** 2020-05-27

**Authors:** Brittany R. Ruhland, Michelle L. Reniere

**Affiliations:** a University of Washington, Department of Microbiology, Seattle, Washington, USA; Ohio State University

**Keywords:** ClpXP, bacterial two-hybrid, disulfide, nitrosative stress, posttranslational regulation, protease adaptor, protein-protein interactions, thioredoxins

## Abstract

Listeria monocytogenes is a model facultative intracellular pathogen. Tight regulation of virulence proteins is essential for a successful infection, and the gene encoding the annotated thioredoxin YjbH was identified in two forward genetic screens as required for virulence factor production. Accordingly, an L. monocytogenes strain lacking *yjbH* is attenuated in a murine model of infection. However, the function of YjbH in L. monocytogenes has not been investigated. Here, we provide evidence that L. monocytogenes YjbH is involved in the nitrosative stress response, likely through its interaction with the redox-responsive transcriptional regulator SpxA1. YjbH physically interacted with SpxA1, and our data support a model in which YjbH is a protease adaptor that regulates SpxA1 protein abundance. Whole-cell proteomics identified eight additional proteins whose abundance was altered by YjbH, and we demonstrated that YjbH physically interacted with each in bacterial two-hybrid assays. Thioredoxin proteins canonically require active motif cysteines for function, but thioredoxin activity has not been tested for L. monocytogenes YjbH. We demonstrated that cysteine residues of the YjbH thioredoxin domain active motif are essential for L. monocytogenes sensitivity to nitrosative stress, cell-to-cell spread in a tissue culture model of infection, and several protein-protein interactions. Together, these results demonstrated that the function of YjbH in L. monocytogenes requires its thioredoxin active motif and that YjbH has a role in the posttranslational regulation of several proteins, including SpxA1.

**IMPORTANCE** The annotated thioredoxin YjbH in Listeria monocytogenes has been implicated in virulence, but its function in the cell is unknown. In other bacterial species, YjbH is a protease adaptor that mediates degradation of the transcriptional regulator Spx. Here, we investigated the function of L. monocytogenes YjbH and demonstrated its role in the nitrosative stress response and posttranslational regulation of several proteins with which YjbH physically interacts, including SpxA1. Furthermore, we demonstrated that the cysteine residues of the YjbH thioredoxin active motif are required for the nitrosative stress response, cell-to-cell spread, and some protein-protein interactions. YjbH is widely conserved among *Firmicutes*, and this work reveals its unique requirement of the thioredoxin-active motif in L. monocytogenes.

## Abstract (precis)

The annotated thioredoxin YjbH in Listeria monocytogenes has been implicated in virulence, but its function in the cell is unknown. In other bacterial species, YjbH is a protease adaptor that mediates degradation of the transcriptional regulator Spx. Here, we investigated the function of L. monocytogenes YjbH and demonstrated its role in the nitrosative stress response and posttranslational regulation of several proteins with which YjbH physically interacts, including SpxA1. Furthermore, we demonstrated that the cysteine residues of the YjbH thioredoxin active motif are required for the nitrosative stress response, cell-to-cell spread, and some protein-protein interactions. YjbH is widely conserved among *Firmicutes*, and this work reveals its unique requirement of the thioredoxin-active motif in L. monocytogenes.

## INTRODUCTION

Bacteria experience abrupt changes in their environment that require immediate adaptation. These perturbations and the ability to respond to them are often life or death situations, such as conditions of nutrient depletion or exposure to deadly reactive oxygen or nitrogen species. Changes at the transcriptional level can be enacted by transcriptional regulators, such as regulators that respond to oxidative stress (1). Adaptation via protein regulation is rapid and can include posttranslational modifications to alter activity, regulated changes in protein solubility, and protease-dependent degradation (2–4).

Exquisite protein regulation is required for the Gram-positive, facultative intracellular pathogen Listeria monocytogenes to navigate the transition from environment to human host. To survive this transition, L. monocytogenes must properly respond to myriad oxidative and nitrosative stressors (5). After the host ingests L. monocytogenes from contaminated food or soil, the pathogen is either engulfed by phagocytic cells or taken up via receptor-mediated endocytosis (6). L. monocytogenes is able to survive the highly oxidative phagosome and escape into the reducing cytosol via the action of the pore-forming toxin listeriolysin O (LLO) (7). Once in the cytosol, L. monocytogenes begins replicating and recruits host actin via ActA, enabling cell-to-cell spread with actin-based motility (8, 9). Each stage of this intracellular life cycle requires tight regulation of virulence proteins. A forward genetic screen in L. monocytogenes for hypohemolytic mutants identified the annotated thioredoxin gene *yjbH* (*yjbH_Lm_*) as being required for LLO secretion and virulence (10). A subsequent screen found *yjbH_Lm_* is also required for ActA production, likely via posttranscriptional regulation of the actA 5′ untranslated region (UTR) (11). Despite the importance of YjbH*_Lm_* to virulence, its function in L. monocytogenes has not been explored.

YjbH is a cytosolic protein with an N-terminal thioredoxin domain and is conserved among *Firmicutes* (10, 12, 13). Much of what is known about YjbH comes from studies on Bacillus subtilis, in which it is a protease adaptor for the ArsC-family redox-responsive transcriptional regulator Spx (14). B. subtilis YjbH (YjbH*_Bs_*) maintains low B. subtilis Spx (Spx*_Bs_*) concentrations during steady state by binding to Spx*_Bs_* and enhancing its ClpXP-mediated degradation (14–16). During disulfide stress, YjbH*_Bs_* aggregation prevents binding to Spx*_Bs_* and, therefore, results in increased Spx*_Bs_* concentrations (17). Spx*_Bs_* is then available to interact with the alpha C-terminal domain of the RNA polymerase to regulate gene expression (18–20). Spx*_Bs_* upregulates over 100 genes, including redox-response genes, such as *trxA* and *trxB*, and represses over 170 genes (21, 22).

The interaction between YjbH*_Bs_* and Spx*_Bs_* has been demonstrated by coimmunoprecipitation and, more recently, the cocrystal structure of Spx*_Bs_* with a thermostable YjbH homologue from Geobacillus kaustophilus (YjbH*_Gk_*) (23, 24). YjbH*_Gk_* is a multidomain protein containing a thioredoxin domain with an alpha-helical insertion and a C-terminal winged-helix domain connected by a linker region (24). Elements of the thioredoxin domain and the alpha-helical insertion are at the interface of the YjbH*_Gk_*-Spx*_Bs_* heterodimer (24). The physical interaction between YjbH*_Bs_* and Spx*_Bs_* is critical to the role of YjbH*_Bs_* as a posttranslational regulator of Spx*_Bs_* (16).

Although all YjbH homologues have a thioredoxin domain and many have the canonical cysteine-X-X-cysteine (CXXC) thioredoxin-active motif, thioredoxin activity has not been demonstrated. The active motif cysteines are essential for thioredoxins to reduce their substrates (25, 26). Both YjbH*_Lm_* and YjbH*_Bs_* have a CXXC motif in the thioredoxin domain, and the Staphylococcus aureus homologue (YjbH*_Sa_*) has SXXC and CXC motifs (27). Cysteine residues are not required for YjbH-mediated degradation of Spx in either B. subtilis or S. aureus (27). The CXXC cysteines and the two cysteines located outside the CXXC motif in YjbH*_Lm_* have never been tested for their contribution to YjbH function.

In this study, we aimed to elucidate the role of YjbH*_Lm_*. L. monocytogenes encodes SpxA1, which is 83% identical in amino acid sequence to Spx*_Bs_*. We showed that YjbH*_Lm_* interacted with SpxA1 and was involved in the nitrosative stress response. Additionally, whole-cell mass spectrometry revealed 10 proteins with increased abundance in a Δ*yjbH_Lm_* mutant. We found that YjbH*_Lm_* physically interacted with nine of these proteins. Interestingly, our work demonstrated that YjbH*_Lm_* uniquely requires its CXXC motif cysteine residues for function, unlike homologues in other species.

## RESULTS

### L. monocytogenes YjbH.

**FIG 1:**
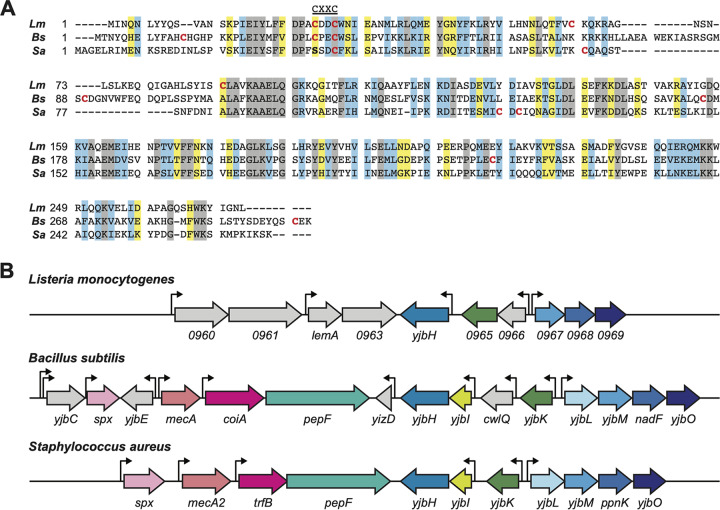
Comparison of YjbH protein sequences and genomic context in Listeria monocytogenes EGD-e, Bacillus subtilis 168, and Staphylococcus aureus NCTC 8325. (A) Amino acid alignment of YjbH homologues (Clustal Omega multiple sequence alignment). Fully conserved residues (gray bars), strongly similar residues (blue bars), and weakly similar residues (yellow bars) are indicated. Cysteine residues are in red. (B) Genomic *yjbH* loci alignment. Predicted transcription start sites are marked with thin black arrows (28, 46, 47). Genes of identical colors encode proteins that are highly similar at the amino acid level (>27% identical), as determined by BLAST. Genes colored gray have no homologues in the pictured loci.

L. monocytogenes encodes a YjbH homologue that shares 39% and 30% amino acid identity with homologues from B. subtilis and S. aureus, respectively (Fig. 1A). All three homologues have a predicted thioredoxin domain at the N terminus and a C-terminal domain of unknown function. A notable difference at the amino acid level between homologues is the presence of cysteine residues. B. subtilis and L. monocytogenes YjbH share a thioredoxin active motif (CXXC), while YjbH*_Sa_* lacks this motif and instead has SXXC and CXC motifs.

The B. subtilis and S. aureus *yjbH* genomic loci are quite conserved with regard to *spx*, the protein product of which is a known interacting partner of YjbH (16, 23). *spx* is encoded near *yjbH* in both B. subtilis and S. aureus (Fig. 1B). Interestingly, while there is synteny downstream of *yjbH_Lm_*, the region upstream of the *yjbH_Lm_* locus is highly dissimilar to B. subtilis and S. aureus. Most notably, *yjbH_Lm_* (*lmo0964*) is not present near the *spx* homologue (*spxA1* and *lmo2191*) as it is in the other two species. These protein similarities and genomic differences led us to question if the function of YjbH is conserved in L. monocytogenes.

### B. subtilis YjbH functionally complements L. monocytogenes Δ*yjbH*.

**FIG 2:**
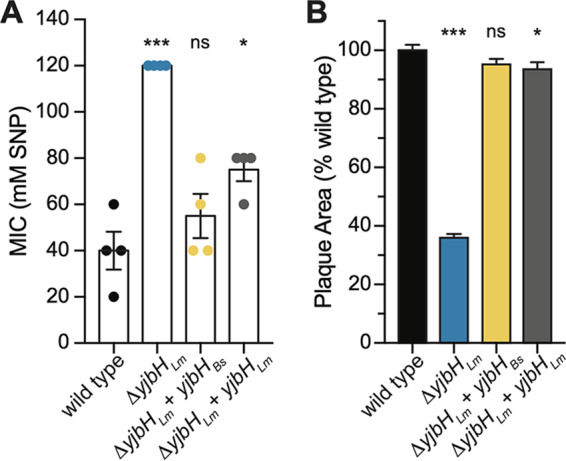
YjbH*_Bs_* functionally complements Δ*yjbH_Lm_* for SNP sensitivity and cell-to-cell spread. (A) Sensitivity to sodium nitroprusside (SNP) was measured by MIC determination in tryptic soy broth at 37°C. Data represent three biological replicates graphed as means and standard error of the mean (SEM). (B) Plaque area in murine L2 cells, normalized to wild-type L. monocytogenes. Data represent three biological replicates graphed as means and SEMs. In both panels, mutant strains are compared to the wild type by Student’s unpaired *t* test (not significant [ns], *P > *0.05; *, *P < *0.05; ***, *P < *0.001).

YjbH*_Bs_* was first identified because the Δ*yjbH_Bs_* mutant was more sensitive to the nitrosative stressor sodium nitroprusside (SNP) (12). Therefore, we first examined the role of YjbH*_Lm_* in L. monocytogenes SNP resistance using an MIC assay that determined growth in 20 mM increments of SNP from 0 to 120 mM. Contrary to what has been shown in both B. subtilis and S. aureus (12, 13), the Δ*yjbH_Lm_* strain was significantly more resistant to SNP than the wild type (Fig. 2A). To test the conservation of YjbH homologues across species, *yjbH_Bs_* was expressed from the predicted *yjbH_Lm_* native promoter at an ectopic locus in the L. monocytogenes Δ*yjbH* genome (28). Expression of *yjbH_Bs_* complemented the Δ*yjbH_Lm_* mutant (Fig. 2A), suggesting that despite the species’ disparate phenotypes, essential functions of YjbH are conserved.

We next tested whether YjbH*_Bs_* could functionally complement the Δ*yjbH_Lm_* mutant during infection by using a plaque assay. Murine fibroblasts were infected, and cell-to-cell spread was measured 3 days postinfection (29). The Δ*yjbH_Lm_* mutant formed plaques approximately 60% smaller than those formed by the wild type, indicating it is defective for cell-to-cell spread, as previously reported (11). The Δ*yjbH_Lm_* strain expressing the *yjbH_Bs_* allele formed plaques approximately the size of the wild type (Fig. 2B), demonstrating that YjbH*_Bs_* is functional in L. monocytogenes during infection of mammalian cells.

### L. monocytogenes YjbH and SpxA1 interact.

In other *Firmicutes*, the physical interaction between YjbH and Spx is critical for the bacteria to maintain redox homeostasis (15, 16, 24). Expression of *yjbH_Bs_* in the Δ*yjbH_Lm_* background restored its SNP sensitivity to wild-type levels, leading us to hypothesize that the interaction with the L. monocytogenes Spx homologue (SpxA1) may also be conserved. We attempted to perform a coimmunoprecipitation to test for interactions between YjbH*_Lm_* and SpxA1. However, because both proteins are present in extremely low abundance in L. monocytogenes and YjbH proteins are prone to aggregation (15, 17), we were unable to detect an interaction. Instead, we used the bacterial adenylate cyclase two-hybrid (BACTH) system, in which proteins of interest are heterologously expressed in Escherichia coli BTH101 cells as fusion proteins with the Bordetella pertussis adenylate cyclase T18 and T25 domains (30, 31). BTH101 strains used in these assays harbor both T18 and T25 fusion protein plasmids, and if the proteins of interest interact, cAMP is produced, leading to β-galactosidase production.

**FIG 3:**
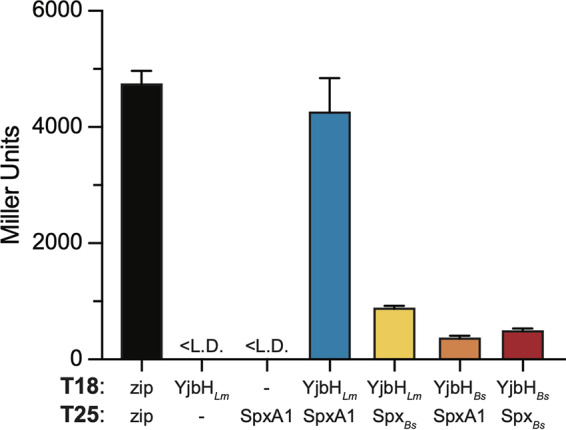
L. monocytogenes YjbH*_Lm_* and SpxA1 physically interact. Interaction was measured by BACTH assay of overnight cultures of LB broth. The positive control is T18 and T25, with each fused to a leucine zipper region, and negative controls are YjbH*_Lm_*-T18 with T25 alone and SpxA1-T25 with T18 alone. Data represent three biological replicates graphed as means and SEMs, with the exception of the SpxA1-T25 with T18 negative control, which represents two biological replicates.

BTH101 cells harboring YjbH*_Lm_*-T18 and SpxA1-T25 expression plasmids grown overnight in rich broth demonstrated that YjbH*_Lm_* and SpxA1 physically interact (Fig. 3). This interaction was specific, as no interaction was detected either between YjbH*_Lm_* and the T25 domain alone or SpxA1 and the T18 domain alone. Because expressing the *yjbH_Bs_* allele functionally complemented the Δ*yjbH_Lm_* mutant, we next investigated interactions between the L. monocytogenes and B. subtilis proteins. We observed that YjbH*_Lm_* interacted with Spx*_Bs_*, and that YjbH*_Bs_* interacted with SpxA1, confirming that this important physical interaction is conserved between L. monocytogenes and B. subtilis proteins (Fig. 3). We also observed an interaction between YjbH*_Bs_* and Spx*_Bs_*, as previously reported (16, 23).

### YjbH*_Lm_* and SpxA1 are involved in the nitrosative stress response and LLO regulation.

**FIG 4:**
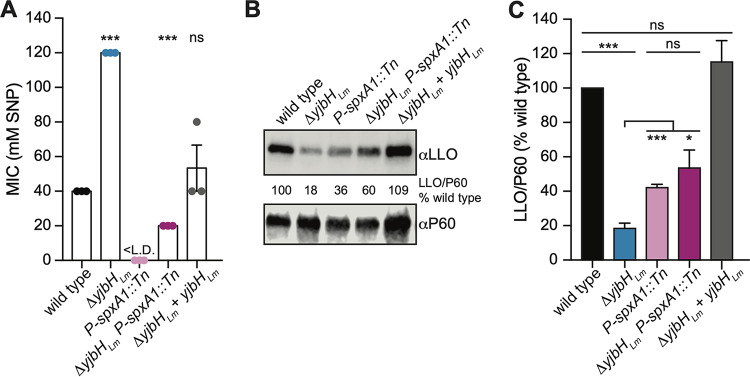
YjbH*_Lm_* and SpxA1 are involved in the nitrosative stress response and LLO regulation. (A) Sensitivity to SNP was measured by MIC in tryptic soy broth at 37°C. The *P-spxA1::*Tn strain transcribes 10-fold less *spxA1* than the wild type and grows aerobically (11). Data represent three biological replicates graphed as means and SEMs. <L.D. indicates the MIC was below the limit of detection. All mutant strains are compared to the wild type by Student’s unpaired *t* test (ns, *P > *0.05; ****, *P < *0.0001). (B) One representative immunoblot of LLO secretion measured in cultures grown in BHI broth at 37°C. The protein P60 was used as a loading control. Immunoblots were analyzed by calculating the ratio of LLO/P60 as a percentage of the wild type. (C) Quantification of three biological replicates of LLO secretion shown in panel B, graphed as means and SEMs. All statistical analyses are Student’s unpaired *t*-tests (ns, *P > *0.05; *, *P < *0.05; ***, *P < *0.001).

The only documented role for YjbH in *Firmicutes* is to modulate levels of Spx such that a strain lacking *yjbH* has increased Spx abundance (14, 16, 27). The physical interaction between YjbH*_Lm_* and SpxA1 was conserved in L. monocytogenes, leading us to question if the phenotypes associated with the *ΔyjbH_Lm_* strain are SpxA1 dependent. The roles of *yjbH_Lm_* and *spxA1* were first examined in an SNP MIC assay. An *spxA1* knockdown strain (*P-spxA1::*Tn strain) that expresses 10-fold less *spxA1* transcript was used for these experiments, as a strain deleted for *spxA1* does not grow in the presence of oxygen (32). The *spxA1* knockdown strain grows aerobically and is more sensitive to hydrogen peroxide and diamide (11) but has not yet been tested in the presence of SNP. While the Δ*yjbH_Lm_* mutant was 3-fold more resistant to SNP, the *P-spxA1::*Tn strain was much more sensitive to SNP than the wild type (Fig. 4A). If the Δ*yjbH* strain was more resistant to SNP due to increased SpxA1 abundance, as in other *Firmicutes*, then knocking down *spxA1* would restore Δ*yjbH_Lm_* sensitivity to wild-type levels. Indeed, the MIC of the Δ*yjbH_Lm_ P-spxA1::*Tn strain was similar to that of the wild type (Fig. 4A). These data suggested that the increased resistance of the Δ*yjbH_Lm_* strain to SNP was due to increased SpxA1 abundance.

In addition to its role in the nitrosative stress response, *yjbH_Lm_* is required for LLO production and/or secretion (10, 11). The virulence factor LLO is essential for L. monocytogenes to escape the phagosome during infection and is also expressed at low levels during growth in rich broth. We examined whether the Δ*yjbH_Lm_* defect in LLO secretion is SpxA1 dependent by analyzing immunoblots of secreted proteins in broth. As previously published (11), both the Δ*yjbH_Lm_* and *P-spxA1::*Tn mutants secreted less LLO than the wild type *in vitro* (Fig. 4B). LLO secretion in the double mutant was not significantly different from the *spxA1* knockdown strain, suggesting that the LLO secretion defect of the Δ*yjbH_Lm_* strain is not solely SpxA1 dependent (Fig. 4C). We hypothesized that the LLO defect in the Δ*yjbH_Lm_* strain happens at the level of translation or secretion. Indeed, hly transcript abundance was unchanged from that of the wild type (1.1-fold change in the Δ*yjbH_Lm_* strain compared to the wild type, *P* = 0.76). The observation that the YjbH*_Lm_*-dependent posttranslational regulation of LLO may not entirely be explained by SpxA1 levels led us to speculate that YjbH*_Lm_* may interact with additional proteins in L. monocytogenes.

### Whole-cell proteomic profiling of L. monocytogenes Δ*yjbH*.

Given the known role of YjbH*_Bs_* as a protease adaptor, we sought to determine whether YjbH*_Lm_* is involved in the regulation of proteins other than SpxA1 in L. monocytogenes. YjbH*_Lm_* abundance was first investigated under various growth conditions to identify ideal parameters for this analysis. In B. subtilis, the abundance of soluble YjbH*_Bs_* is affected by stressors, such as ethanol, diamide, and heat (17). However, native YjbH*_Lm_* was undetectable under all conditions tested, including elevated temperature and treatment with sublethal concentrations of ethanol, diamide, SNP, or hydrogen peroxide (see Fig. S1 in the supplemental material). Therefore, early stationary phase was selected for proteomic analysis due to the pronounced LLO phenotype exhibited by the Δ*yjbH_Lm_* strain in rich broth at this time point (Fig. 4B and C), indicating that YjbH*_Lm_* is likely functional under this growth condition. Whole-cell proteomic profiling was used as an unbiased approach to identify proteins with altered abundance in the Δ*yjbH_Lm_* strain. Cultures were prepared in biological triplicate, and proteins were analyzed by liquid chromatography-tandem mass spectrometry (LC-MS/MS). The fold change of average peptide spectral counts was calculated between wild-type and Δ*yjbH_Lm_* samples and analyzed with Student’s *t* test (see Data Set S1 in the supplemental material).

**FIG 5:**
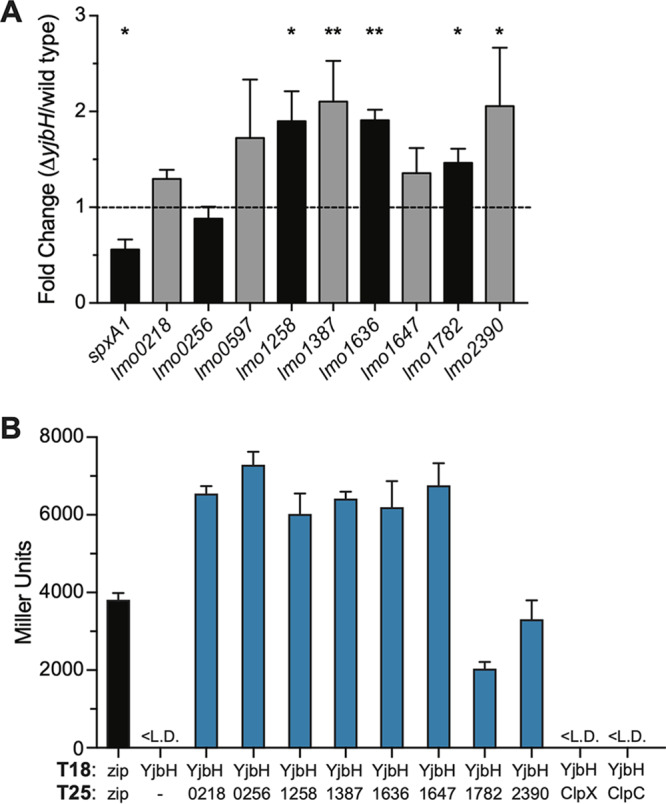
YjbH*_Lm_* interacts with multiple L. monocytogenes proteins. (A) Gene expression was measured in wild-type and Δ*yjbH_Lm_* strains by quantitative RT-PCR and is graphed as the fold change in the Δ*yjbH_Lm_* strain compared to wild type. Student’s unpaired *t* test was used to compare transcript fold changes between the Δ*yjbH_Lm_* strain and wild type (*, *P < *0.05; **, *P < *0.01). (B) Interactions with YjbH*_Lm_* were measured by BACTH assay of overnight cultures of LB broth. T25 fusion proteins are labeled with their *lmo* locus number or protein name. Data represent three biological replicates graphed as means and SEMs. <L.D. indicates Miller units below the limit of detection.

We focused on the nine proteins significantly more abundant in the Δ*yjbH_Lm_* strain than the wild type (Table 1). SpxA1 was also more abundant in the Δ*yjbH_Lm_* strain, although this change was not statistically significant due to its complete absence in wild-type samples and the variability of peptide counts between Δ*yjbH_Lm_* replicates. These data demonstrated for the first time that SpxA1*_Lm_* is more abundant in the absence of *yjbH_Lm_*, supporting the hypothesis that YjbH*_Lm_* functions similarly to YjbH*_Bs_* with respect to regulating SpxA1 abundance (14, 16). To assess whether the observed changes in protein concentration were the result of transcriptional or posttranscriptional regulation, quantitative reverse transcriptase PCR (RT-PCR) was performed comparing gene expression in wild-type and Δ*yjbH_Lm_* cultures (Fig. 5A). Five transcripts (*lmo1258*, *lmo1387*, *lmo1636*, *lmo1782*, and *lmo2390*) were significantly more abundant in the Δ*yjbH_Lm_* strain than in the wild type, suggesting that increased protein concentration may result from transcriptional regulation. It is possible that these five genes are also posttranscriptionally regulated to result in increased protein levels. Five genes (*spxA1*, *lmo0218*, *lmo0256*, *lmo0597*, and *lmo1647*) were expressed at or below wild-type levels in the Δ*yjbH_Lm_* strain, indicating that the increases in protein abundance observed by mass spectrometry were not due to transcriptional regulation. These results suggested that YjbH*_Lm_* is involved in the posttranscriptional or posttranslational regulation of multiple proteins in L. monocytogenes.

**TABLE 1:** Whole-cell proteomics revealed proteins more abundant in the Δ*yjbH_Lm_* strain than in the wild type

Gene locus	Predicted function	Protein	Avg spectral peptide count^*a*^ of:		*P* value
			Wild type	Δ*yjbH_Lm_* strain	
*lmo0218*	Polyribonucleotide nucleotidyltransferase domain		1.53	3.68	0.006
*lmo0256*	*S*-Adenosylmethionine-dependent methyltransferase activity		1.22	3.00	0.045
*lmo0597*	Crp/Fnr-family transcriptional regulator		0.31	2.24	0.006
*lmo1258*	Putative lipase		ND	2.82	0.028
*lmo1387*	Pyrroline-5-carboxylate reductase		0.61	2.68	0.045
*lmo1636*	ABC transporter ATP-binding protein		2.14	4.47	0.011
*lmo1647*	1-Acylglycerol-3-phosphate *O*-acyltransferase		0.61	2.56	0.007
*lmo1782*	3′-Exo-deoxyribonuclease		1.23	3.93	0.009
*lmo2191*	Transcriptional regulator	SpxA1	ND	2.76	0.214
*lmo2390*	Ferredoxin-NADP reductase 2		2.45	5.94	0.005

a Data are from three independent samples. ND, no peptides were detected.

### YjbH interacts with multiple L. monocytogenes proteins.

BACTH assays were next used to test the hypothesis that YjbH*_Lm_* function in L. monocytogenes involves direct interaction with proteins in addition to SpxA1. BTH101 strains were generated that each harbored a plasmid expressing YjbH*_Lm_*-T18 in addition to a plasmid expressing a T25 fusion with each of the proteins listed in Table 1. We were unable to express *lmo0597* in E. coli and, therefore, could not test for an interaction with YjbH*_Lm_*. Interestingly, BACTH assays revealed a physical interaction between YjbH*_Lm_* and each of the eight proteins identified by mass spectrometry as more abundant in the Δ*yjbH_Lm_* strain (Fig. 5B). Some chaperone proteins are known to interact with the ATPase and substrate-binding subunit of the protease as well as with the protein substrate (33). ClpC and ClpX ATPase subunits have both been implicated in Spx*_Bs_* degradation in B. subtilis, although YjbH*_Bs_*-mediated Spx*_Bs_* degradation is specific to ClpXP (16, 34). However, we were unable to detect an interaction between YjbH*_Lm_* and ClpC or ClpX by BACTH (Fig. 5B). Together, these results indicated that YjbH*_Lm_* is able to physically interact with at least nine L. monocytogenes proteins, including SpxA1.

### YjbH cysteine residues influence function and protein-protein interactions.

Although all YjbH homologues have a thioredoxin-like domain and many have a CXXC catalytic motif (Fig. 1A), it remains unknown whether thioredoxin activity is important for YjbH function in L. monocytogenes. Canonical thioredoxin activity involves a transient intermolecular disulfide bond between the thioredoxin CXXC motif and substrate proteins. To test the role of the four cysteine residues of YjbH*_Lm_*, Δ*yjbH_Lm_* strains were engineered to express *yjbH_Lm_* encoding cysteine-to-alanine point mutations from the predicted *yjbH_Lm_* native promoter (see Table S1 in the supplemental material). These strains include each of the four cysteines mutated individually (YjbH^C27A^, YjbH^C30A^, YjbH^C63A^, and YjbH^C89A^), as well as a mutated CXXC motif (YjbH^C27/30A^), both cysteines outside the CXXC motif mutated (YjbH^C63/89A^), and all four cysteine residues mutated (YjbH^Δcys^). The mutant YjbH*_Lm_* proteins were as abundant as wild-type YjbH*_Lm_* when overexpressed (see Fig. S2 in the supplemental material), indicating that the cysteine-to-alanine substitutions did not affect overall protein stability.

**FIG 6:**
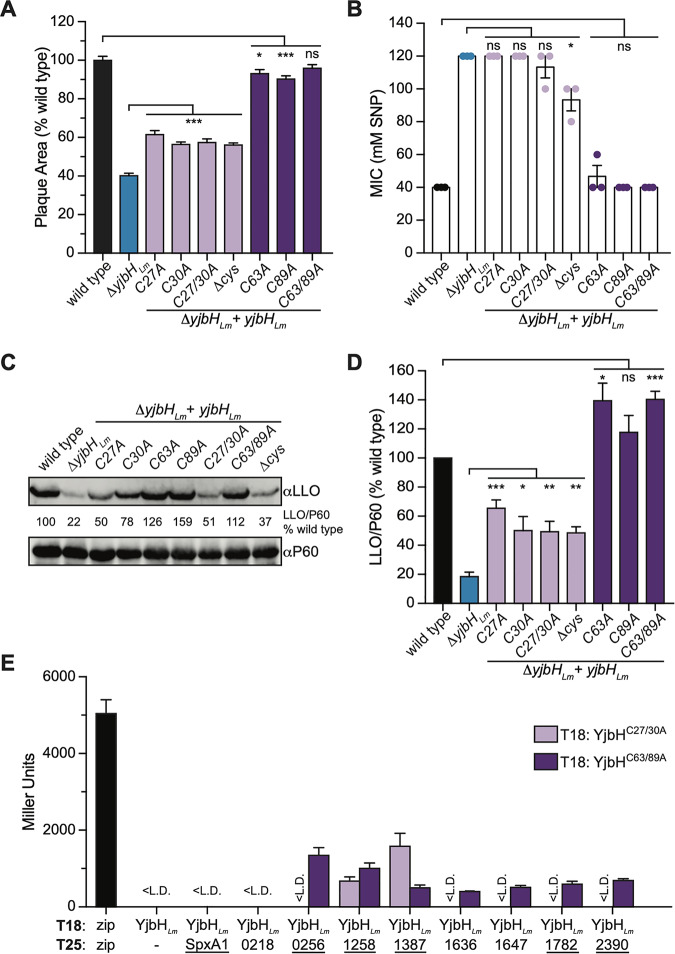
Cysteine residues contribute to YjbH*_Lm_* function. (A) Plaque area in murine L2 cells, normalized to wild-type L. monocytogenes. Data represent three biological replicates graphed as means and SEMs. (B) Sensitivity to SNP was measured by MIC determination in tryptic soy broth at 37°C. Data represent three biological replicates graphed as means and SEMs. (C) One representative immunoblot of LLO secretion, measured as in Fig. 4C. (D) Graphed data represent three biological replicates of LLO secretion shown in panel C, graphed as means and SEMs. (E) Interactions with YjbH^C27/30A^ and YjbH^C63/89A^ were by determined by BACTH assay as in Fig. 5B. Gene names that are underlined encode proteins with at least one cysteine residue. <L.D. indicates Miller units below the limit of detection. Data represent three biological replicates graphed as means and SEMs. In panels A, B, and D, Student's *t* test was used to compare lavender bar data to data for the Δ*yjbH_Lm_* strain and dark purple bar data to data for the wild type (ns, *P > *0.05; *, *P < *0.05; **, *P < *0.01; ***, *P < *0.001).

To investigate the role of the cysteine residues in YjbH*_Lm_* function, the aforementioned mutants were tested for cell-to-cell spread by plaque assay, nitrosative stress resistance via SNP MIC assay, and LLO secretion by immunoblot. The data revealed that YjbH^C63A^, YjbH^C89A^, and YjbH^C63/89A^ fully complemented the Δ*yjbH_Lm_* mutant for cell-to-cell spread (Fig. 6A, dark purple bars), SNP resistance (Fig. 6B), and LLO secretion (Fig. 6C and D). However, all strains with mutations in the CXXC motif failed to fully rescue the Δ*yjbH_Lm_* phenotypes (Fig. 6A to D, lavender bars). These data suggested that the CXXC motif is required for YjbH*_Lm_* function in the context of the known phenotypes, while the other two cysteine residues are dispensable.

We next used BACTH assays to assess whether the YjbH*_Lm_* cysteine residues are required for protein-protein interactions. YjbH^C63/89A^ retained interactions with all tested proteins except SpxA1 and Lmo0218 (Fig. 6E). The protein with an altered CXXC motif (YjbH^C27/30A^) interacted only with Lmo1258 and Lmo1387 (Fig. 6E). Together, these results demonstrated that YjbH*_Lm_* cysteine residues are required for its physical interaction with some proteins.

As described previously, the CXXC motifs of thioredoxin domains are known to form transient intermolecular disulfide bonds with substrate proteins. Of those tested here, only the following proteins contain cysteine residues: SpxA1, Lmo0256, Lmo1258, Lmo1387, Lmo1782, and Lmo2390 (Fig. 6E, underlined). If intermolecular disulfide bonds involving the CXXC motif were essential for YjbH*_Lm_* to interact with any of the cysteine-containing proteins, we would expect the interacting proteins to bind the wild-type YjbH*_Lm_* protein but not the YjbH^C27/30A^ mutant. The observation that YjbH^C27/30A^ could still bind two proteins suggested that YjbH*_Lm_* can interact with proteins at a site other than the CXXC motif. These results demonstrated that not all YjbH*_Lm_* protein interactions require intermolecular disulfide bond formation, as is typical for thioredoxins. However, we cannot rule out a role for disulfide bonds in all interactions.

## DISCUSSION

In this study, we investigated the role of the annotated thioredoxin YjbH*_Lm_* in L. monocytogenes. Strains lacking *yjbH_Lm_* do not have a growth defect intracellularly or in rich broth but exhibit defects in cell-to-cell spread, the production of ActA and LLO, and are attenuated in a mouse model of infection (11). Our results demonstrated that YjbH*_Lm_* physically interacts with the redox-responsive transcriptional regulator SpxA1 and that the Δ*yjbH_Lm_* strain is more resistant to nitrosative stress than the wild type in an SpxA1-dependent manner. This study also presented data that suggest a possible SpxA1-independent role for YjbH*_Lm_* in LLO secretion. Whole-cell proteomics provided a more holistic picture of the scope of YjbH*_Lm_* function, revealing several proteins whose abundance was YjbH*_Lm_* dependent. Furthermore, eight proteins that were more abundant in the Δ*yjbH_Lm_* strain physically interacted with YjbH*_Lm_* in BACTH assays. The interactions of YjbH*_Lm_* with SpxA1 and six other proteins were disrupted in the absence of an intact YjbH*_Lm_* CXXC motif. Results from this study demonstrated that YjbH*_Lm_* requires its thioredoxin active motif for SNP sensitivity, cell-to-cell spread, and LLO secretion and that YjbH*_Lm_* plays a role in the posttranslational regulation of several proteins.

In B. subtilis, YjbH*_Bs_* physically interacts with Spx*_Bs_* to accelerate degradation of Spx*_Bs_* by the ClpXP protease. The results presented here suggest that YjbH*_Lm_* functions similarly as a protease adaptor for SpxA1. First, BACTH assays demonstrated a physical interaction between YjbH*_Lm_* and SpxA1. YjbH*_Lm_* did not interact with the protease substrate-binding subunits ClpC or ClpX, although this was consistent with YjbH*_Bs_*, which does not physically interact with ClpX (15). Instead, YjbH*_Bs_* acts as an adaptor by lowering the conformational entropy of Spx*_Bs_*, thereby increasing the efficiency of Spx*_Bs_* binding to ClpX (24). We predict this mechanism is conserved in L. monocytogenes, as SpxA1 shares 83% amino acid identity with Spx*_Bs_* and expressing *spx_Bs_* functionally complements the *ΔspxA1* mutant (32). The second piece of evidence that YjbH*_Lm_* is a protease adaptor for SpxA1 comes from whole-cell proteomics, which revealed increased SpxA1 protein levels in the Δ*yjbH_Lm_* strain. Although protein levels were increased, spxA1 transcript abundance was decreased in the Δ*yjbH_Lm_* strain, indicating YjbH*_Lm_*-dependent posttranscriptional regulation. Finally, the Δ*yjbH_Lm_* strain with increased SpxA1 abundance had a corresponding increase in SNP resistance, demonstrating that the response to nitrosative stress in L. monocytogenes is SpxA1 dependent. Taken together, these results support a model in which YjbH*_Lm_* is a protease adaptor that regulates SpxA1 protein abundance in L. monocytogenes.

It is interesting to note that the L. monocytogenes *ΔyjbH* mutant was more resistant to nitrosative stress, while the B. subtilis and S. aureus *ΔyjbH* mutants are more sensitive (12, 35). However, expressing the YjbH*_Bs_* protein in the Δ*yjbH_Lm_* strain fully restored its sensitivity to SNP. While these results may seem counterintuitive at first, we propose that the nitrosative stress phenotypes of bacteria lacking *yjbH* are dependent on factors regulated by Spx. We demonstrated that both YjbH*_Lm_* and YjbH*_Bs_* interact with SpxA1 and will, therefore, similarly affect the expression of SpxA1-dependent proteins. Defining the L. monocytogenes SpxA1 regulon is the next step to more completely define the differences between the organisms with respect to the differing YjbH-dependent responses to nitrosative stress.

Regulation of LLO production, secretion, and activity is complex and incompletely understood in L. monocytogenes (36, 37). YjbH*_Lm_* was found to play a role in LLO regulation over a decade ago; yet, the mechanism behind this phenotype remains to be elucidated. We demonstrated here that the Δ*yjbH_Lm_* defect in LLO secretion is likely partially SpxA1 independent. The Δ*yjbH_Lm_* strain, which has more SpxA1 than the wild type, is severely deficient in LLO secretion. Conversely, the *spxA1* knockdown strain is also deficient in LLO. Together, these data revealed that LLO production is partially regulated in an SpxA1-dependent manner. Although SpxA1 is a transcriptional regulator and the *hly* transcript is unchanged, this regulation could be through posttranscriptional effects of the SpxA1 regulon. However, the fact that deleting *yjbH_Lm_* in the *spxA1* knockdown strain did not rescue the LLO secretion defect suggests that part of this phenotype may be YjbH*_Lm_* dependent and SpxA1 independent. No other studies on a YjbH homologue have presented evidence of Spx-independent functions of YjbH.

To elucidate YjbH*_Lm_* function in an unbiased manner, we performed whole-cell proteomics on the Δ*yjbH_Lm_* strain. In this study, we focused on proteins that were more abundant in the absence of *yjbH_Lm_*, due to the known role of YjbH*_Bs_* as a protease adaptor. With the exception of SpxA1, we concentrated on proteins whose increased abundance was statistically significant. It is possible that more proteins on this list are biologically significant but fall outside the bounds of statistical significance. There was also an extensive list of proteins significantly less abundant in the Δ*yjbH_Lm_* strain than the wild type (see Table S2 in the supplemental material). While the study of these proteins was outside the scope of this work, future work will investigate how YjbH*_Lm_* is capable of increasing the concentration of proteins like LLO when its only known role is that of a protease adaptor. Even more intriguing is the fact that YjbH*_Lm_* regulates two transcription factors, namely SpxA1 and Lmo0597. The regulons of SpxA1 and Lmo0597 have not been reported, and thus, it is unknown how altering levels of SpxA1 and Lmo0597 may contribute to Δ*yjbH_Lm_* phenotypes. Of the five genes significantly increased in expression in the Δ*yjbH_Lm_* strain, only one has a homologue in B. subtilis that is regulated by Spx*_Bs._* The putative 3′-exo-DNase *lmo1782* is 69% identical to *exoA* and upregulated by Spx*_Bs_* during diamide stress (21). Ongoing experiments are aimed at deciphering the downstream effects of YjbH-dependent regulation of SpxA1 and Lmo0597.

Protease adaptor activity is dependent on direct interactions between the adaptor and substrate proteins. In BACTH assays with L. monocytogenes proteins, we found that YjbH*_Lm_* interacted with 8 out of 10 tested proteins in addition to SpxA1. This finding was intriguing, as the only characterized YjbH*_Bs_* interacting partners are Spx*_Bs_* and the small inhibitor protein YirB, which is not conserved among *Firmicutes* and is not present in L. monocytogenes. An unbiased yeast two-hybrid screen of a B. subtilis genomic fusion library detected seven additional proteins that interacted with YjbH*_Bs_* (23). Thus, while there is precedent for YjbH*_Bs_* to bind proteins other than Spx*_Bs_*, it is not known if any of these uncharacterized binding partners contribute to YjbH*_Bs_* function. In L. monocytogenes, whole-cell proteomics showed that SpxA1 and the eight uncharacterized interacting partners were more abundant in the Δ*yjbH_Lm_* strain, raising the possibility that YjbH*_Lm_* posttranslationally regulates other proteins in addition to SpxA1. From their predicted functions, it was not obvious what these interacting partners have in common or what roles they may play in the cell. However, the ability of YjbH*_Lm_* to interact with such a wide variety of proteins will inform future work to explain the myriad phenotypes exhibited by L. monocytogenes Δ*yjbH_Lm_*.

YjbH homologues share a conserved thioredoxin domain at the N terminus. A CXXC motif is required for catalytic activity in thioredoxin family proteins, but YjbH*_Bs_* and YjbH*_Sa_* do not require CXXC motif cysteines for function (17, 27). We demonstrated here that the cysteine residues of the YjbH*_Lm_* CXXC motif were required to fully complement a Δ*yjbH_Lm_* mutant for SNP sensitivity, LLO secretion, and cell-to-cell spread. However, the YjbH*_Lm_* protein with a mutated CXXC motif still retained partial functionality, perhaps through protein-protein interactions at a site other than the CXXC motif. For example, two out of nine proteins (Lmo1258 and Lmo1387) were still able to interact with YjbH^C27/30A^, which lacks a functional CXXC motif. The two cysteine residues located outside the CXXC motif were required for interacting with SpxA1 and Lmo0218, although C63 and C89 were dispensable for YjbH function during cell-to-cell spread and SNP stress. In B. subtilis, the YjbH*_Bs_* cysteine residues are not required for enhancing Spx*_Bs_* degradation or for autoaggregation (17, 27). Cysteines are also dispensable to YjbH*_Sa_* function in virulence and Spx*_Sa_* degradation (27, 35). YjbH*_Sa_* does not contain a CXXC motif but instead has an SXXC motif that aligns with the L. monocytogenes and B. subtilis N-terminal CXXC motif and a CXC motif at nucleotide position 114 to 116 (27, 35). The lack of conservation of the CXXC motif across all YjbH homologues and the apparent lack of conserved cysteine essentiality suggest either that thioredoxin activity is not necessary for function or that CXXC motifs have disparate functions between species.

This is the first work to characterize YjbH*_Lm_* and will serve as a foundation for future studies aimed at uncovering the full scope of its role. YjbH*_Sa_* transcomplements Δ*yjbH_Bs_* (27), and we have shown here that YjbH*_Bs_* complements the L. monocytogenes mutant. Together with our findings that Δ*yjbH_Lm_* results in increased SpxA1 abundance, which indicates that YjbH*_Lm_* is posttranslationally regulating SpxA1, this demonstrates conserved function between *Firmicutes*. However, YjbH*_Lm_* has unique characteristics that set it apart from other homologues. For example, YjbH*_Lm_* is present in very low abundance under every growth condition examined, the LLO secretion defect is at least partially SpxA1 independent, YjbH*_Lm_* has a role in the posttranscriptional regulation of at least nine proteins, and YjbH*_Lm_* requires the CXXC motif cysteines for function. The basic characterization presented here is, thus, both broadly applicable to other *Firmicutes* species and a step forward in understanding the uniquely complex role of YjbH*_Lm_* in L. monocytogenes.

## MATERIALS AND METHODS

### Bacterial strains and culture conditions.

All L. monocytogenes strains are a derivative of strain 10403S (38, 39). L. monocytogenes strains were cultivated in brain heart infusion broth (BHI; Difco) or tryptic soy broth (TSB; Difco) shaking at 37°C, and E. coli strains were cultivated in LB broth (Miller) shaking at 37°C. Antibiotics were used at the following concentrations for Gram-negative strains: 100 μg/ml (carbenicillin) and 50 μg/ml (kanamycin). Antibiotics were used at the following concentrations for Gram-positive strains: 200 μg/ml (streptomycin), 5 μg/ml (chloramphenicol), and 2 μg/ml (tetracycline).

### Cloning and plasmid construction.

The integrating plasmid pPL2 was used to generate L. monocytogenes mutants, as previously described (40). Briefly, genes of interest were amplified from L. monocytogenes 10403S and digested with the same restriction endonucleases (New England BioLabs) used to digest the pPL2 vector, followed by a ligation reaction to join the insert with the vector. Ligation products were transformed into E. coli SM10 and sequenced before proceeding.

Constructed plasmids in E. coli SM10 were transconjugated into recipient L. monocytogenes strains by mixing donor and recipient cells 1:1 and incubating on BHI agar plates for 4 to 24 h at 30°C. Cell mixtures were then streaked onto BHI plates containing streptomycin and chloramphenicol to select for cells containing the pPL2 plasmid. Resulting colonies were restreaked for isolation and sequenced before use.

The Δ*yjbH P-spxA1::*Tn strain was constructed by transducing the *himar1* transposon into the Δ*yjbH* background, as previously described (10, 41).

Plasmids for the BACTH assays were constructed via Gibson assembly using the NEBuilder HiFi DNA assembly master mix (New England BioLabs). Briefly, genes of interest were amplified with 5′ (ATGGGGTCCAGCGGCGCTGGATCC) and 3′ (GCTGCAGGAGGCAGTGGAGCGAGC) linker regions identical to linker regions flanking a *ccdB* toxin cassette in the pUT18x, pUT18Cx, pKT25x, and pKNT25x vectors, which were generously gifted to us by Aaron Whiteley (42). Vectors were linearized with BamHI and PstI endonucleases that excised the *ccdB* cassette and then were combined in the NEB master mix with an insert encoding the gene of interest, as directed by the manufacturer. The reaction mix was transformed into E. coli XL1-Blue cells. XL1-Blue cells containing single BACTH vectors were sequenced, and their plasmid DNA was harvested. Each BTH101 strain used in BACTH assays was cotransformed with one T18 plasmid and one T25 plasmid. After cotransformations, all BTH101 strains were cultivated in both kanamycin and carbenicillin at all times to retain both plasmids.

### MIC assays.

L. monocytogenes cultures were grown overnight in TSB medium containing streptomycin at 37°C. Assays were performed in 96-well plates. Each well contained 200 μl total of TSB medium, SNP (solution made fresh daily in TSB with streptomycin), and 2 × 10^4^ CFU. Each L. monocytogenes strain was tested in 120 mM, 100 mM, 80 mM, 60 mM, 40 mM, 20 mM, 10 mM, and 0 mM SNP. Overnight cultures were diluted in phosphate-buffered saline (PBS) to 10^4^ CFU/ml before being added to assay wells. Plates were incubated in shaking plate incubator at 37°C for 24 h, and then the MIC for each strain was determined as the lowest concentration of SNP with no visible bacterial growth.

### L2 plaque assays.

Plaque assays were performed according to published protocols (29). Briefly, L2 fibroblasts were seeded at a density of 1.2 × 10^6^ per well in a 6-well dish, and overnight cultures of L. monocytogenes were incubated at 30°C in BHI broth. The next day, cultures were diluted 1:10 in sterile PBS and 5 μl was used to infect cells for 1 h before being washed twice with sterile PBS. Agarose overlays containing Dulbecco’s modified Eagle’s medium (DMEM) and gentamicin were added to the wells, and plates were incubated for 2 days before the cells were stained with neutral red dye. Plaques were imaged 24 h later, and plaque areas were determined using Image J software (43). All plaque data represent three biological replicates.

### Immunoblotting for the LLO protein.

Overnight L. monocytogenes cultures were subcultured 1:10 into BHI medium containing streptomycin and were incubated for 5 h at 37°C with shaking. Cultures were pelleted, and the supernatant was then trichloroacetic acid (TCA)-precipitated to collect protein, boiled in loading dye, and separated by gel electrophoresis. Proteins were then transferred to a polyvinylidene difluoride (PVDF) membrane (Bio-Rad), and the membrane was blocked with Odyssey blocking buffer (Li-Cor Biosciences). Proteins of interest were detected using polyclonal rabbit anti-LLO antibody (from the Portnoy Laboratory, University of California at Berkeley) at a dilution of 1:5,000 and monoclonal mouse anti-P60 antibody (Adipogen) at a dilution of 1:2,000. Goat anti-rabbit (Invitrogen) and goat anti-mouse (Li-Cor Biosciences) antibodies were used to detect the primary antibodies, each at a dilution of 1:5,000. Immunoblots were imaged on a Li-Cor Odyssey Fc system and were analyzed using Image Studio software to calculate band densitometry. All immunoblots were performed in biological triplicates.

### Bacterial two-hybrid broth quantification.

E. coli BTH101 strains, each containing one pUT18x vector (carbenicillin resistance) and one pKT25x vector (kanamycin resistance), were grown overnight at 37°C in LB broth containing kanamycin and carbenicillin. Cultures were permeabilized by combining 800 μl Z-Buffer, 20 μl 0.1% SDS, 40 μl chloroform, and 200 μl overnight culture. The assay was performed in a 96-well plate, and each strain was assayed in technical triplicates. Each well contained 150 μl Z-Buffer, 50-μl permeabilized culture solution, and 40 μl 0.4% *o*-nitrophenyl-β-d-galactopyranoside (ONPG) to start the reaction. *A*_420_ and *A*_550_ values were collected every 2 minutes for 30 minutes at 28°C. *A*_420_ data were graphed to determine the linear portion of the reaction, from which the calculations were performed. Miller units were calculated with the following equation: [(*A*_420_ − (1.75 × *A*_550_))/(*t* × *v* × *A*_600_)] × 1,000 where *t* equals time in minutes and *v* equals culture volume used in the assay in milliliters (44). All BACTH data represent three biological replicates.

### Whole-cell proteomics sample preparation.

Wild-type and Δ*yjbH* L. monocytogenes were grown overnight and then subcultured for 5 h shaking at 37°C in BHI broth containing streptomycin. Cultures were then centrifuged for 20 minutes at 4°C. Pellets were sonicated in lysis buffer (0.1 M ammonium bicarbonate, 8 M urea, and 0.1% [wt/vol] Rapigest detergent [Waters]) and centrifuged at maximum speed. Tris(2-carboxyethyl)phosphine hydrochloride (TCEP) was added to supernatant fractions to a final concentration of 5 mM. Samples were incubated at room temperature for 1 hour before adding iodoacetamide to 10 mM. Samples were then incubated in the dark at room temperature for 30 minutes before adding *N*-acetylcysteine to 15 mM. Samples were then digested with trypsin gold (Promega) overnight at 37°C by combining 200 μg of each sample (determined by BCA assay) with trypsin in a 1:20 (wt/wt) (trypsin/protein) ratio. Following trypsinization, 2 M HCl was added dropwise to each sample until samples became acidic (pH 1 to 2), as monitored with pH paper. Detergent was removed by centrifugation at 10,000 × *g* for 5 minutes, and the supernatant was transferred into new tubes. Acetonitrile (ACN) and trifluoroacetic acid (TFA) were added to 5% and 0.1% (vol/vol), respectively. Samples were processed through MacroSpin C_18_ columns (30- to 300-μg capacity; The Nest Group) as directed and washed with 5% ACN and 0.1% TFA. Samples were eluted with 80% ACN and 25 mM formic acid. Samples were then sent to the Northwestern University Proteomics Core for analysis by mass spectrometry. The sample preparation protocol is adapted from the Mougous Laboratory at the University of Washington Department of Microbiology (45).

### LC-MS/MS analysis.

Peptides were analyzed by LC-MS/MS using a Dionex UltiMate 3000 rapid separation nanoLC instrument and an Orbitrap Elite mass spectrometer (Thermo Fisher Scientific, Inc., San Jose, CA). Samples were loaded onto the trap column, which was 150 μm by 3 cm, and in-house packed with 3-μm ReproSil-Pur beads. The analytical column was a 75-μm by 10.5-cm PicoChip column packed with 3-μm ReproSil-Pur beads (New Objective, Inc., Woburn, MA). The flow rate was kept at 300 nl/min. Solvent A was 0.1% formic acid (FA) in water and solvent B was 0.1% FA in ACN. The peptide was separated on a 120-min analytical gradient from 5% ACN/0.1% FA to 40% ACN/0.1% FA. MS^1^ scans were acquired from 400 to 2,000 *m/z* at a 60,000 resolving power and with the automatic gain control (AGC) set to 1 × 10^6^ intensity. The 15 most abundant precursor ions in each MS^1^ scan were selected for fragmentation by collision-induced dissociation (CID) at 35% normalized collision energy in the ion trap. Previously selected ions were dynamically excluded from reselection for 60 seconds.

### Data analysis.

Proteins were identified from the MS raw files using the Mascot search engine (Matrix Science, London, UK; version 2.5.1). MS/MS spectra were searched against the UniProt Listeria monocytogenes database. All searches included carbamidomethyl cysteine as a fixed modification and oxidized Met, deamidated Asn and Gln, and acetylated N-term as variable modifications. Three missed tryptic cleavages were allowed. The MS^1^ precursor mass tolerance was set to 10 ppm and the MS^2^ tolerance was set to 0.6 Da. The search result was visualized by Scaffold (version 4.8.3; Proteome Software, Inc., Portland, OR). A 1% false discovery rate cutoff was applied at the peptide level. Only proteins with a minimum of two unique peptides above the cutoff were considered for further study.

### Quantitative RT-PCR of bacterial transcripts.

Overnight cultures of wild-type and Δ*yjbH* L. monocytogenes were diluted 1:100 into 25 ml of BHI in a 250-ml flask and grown in BHI broth at 37°C shaking until cultures reached an optical density at 600 nm (OD_600_) of 1.0. At this point, 5 ml of each culture was pipetted into 5 ml ice-cold MeOH, inverted to mix, and centrifuged at 4°C to pellet. All supernatant was removed, and pellets were frozen in liquid nitrogen before storing at –80°C overnight.

Frozen pellets were resuspended in 400 μl AE buffer (50 mM sodium acetate [NaOAc; pH 5.2] and 10 mM EDTA, diethyl pyrocarbonate [DEPC] treated and autoclaved) and vortexed vigorously. Samples were kept on ice as much as possible during entire protocol. A total of 400 μl of the resuspended pellet mixture was transferred to an RNase-free 1.5-ml tube on ice containing 50 μl 10% SDS and 500 μl 1:1 acidified phenol-chloroform (made fresh, with aqueous layer removed before addition of the pellet mixture). Samples were then lysed by bead beating and returned to ice. Lysed samples were applied to prespun 5Prime phase lock gel heavy tubes (Quantabio) and centrifuged at maximum speed for 5 minutes. The aqueous layer was pipetted into a new 1.5-ml tube containing 50 μl 3 M NaOAc at pH 5.2 and 1 ml 100% ethanol (EtOH) and was vortexed to mix. Samples were centrifuged at 4°C for 30 minutes at maximum speed, at which point the EtOH was removed by aspiration. A total of 500 μl 70% EtOH was added to each sample and vortexed to mix. After centrifuging at room temperature for 10 minutes at maximum speed, the EtOH was removed by aspiration and then by running samples in a speed vac. Samples were resuspended in 50 μl water and reverse transcribed using an iScript cDNA synthesis kit (Bio-Rad). Quantitative RT-PCR was performed on cDNA with the iTaq universal SYBR green supermix (Bio-Rad).

## Supplementary Material

Supplemental file 1

Supplemental file 2
