# Supplementary material for: YjbH Requires Its Thioredoxin Active Motif for the Nitrosative Stress Response, Cell-to-Cell Spread, and Protein-Protein Interactions in Listeria monocytogenes
Source: J Bacteriol. 2020 May 27;202(12):e00099-20. doi: 10.1128/JB.00099-20 (PMC7253607; doi:10.1128/JB.00099-20)
Supplement: Supplemental file 1 [file JB.00099-20-s0002.pdf]

SUPPLEMENTARY INFORMATION

YjbH requires its thioredoxin active motif for the nitrosative stress response, cell-to-cell spread, and protein-protein interactions in *Listeria monocytogenes*

Brittany R. Ruhland[a] and Michelle L. Reniere[a#]

[a]University of Washington Department of Microbiology

[#]reniere@uw.edu

CONTENTS

Figure S1. Natively expressed YjbH_{Lm} is undetectable by immunoblot.

Figure S2. YjbH_{Lm} cysteine mutants are equally as stable as wild type YjbH_{Lm}.

Table S1. *Listeria monocytogenes* strains used in this study.

Table S2. Whole-cell proteomics revealed proteins less abundant in Δ*yjbH* than wild type.

Table S3. *E. coli* strains used in this study.

Supplementary Methods

Supplementary References

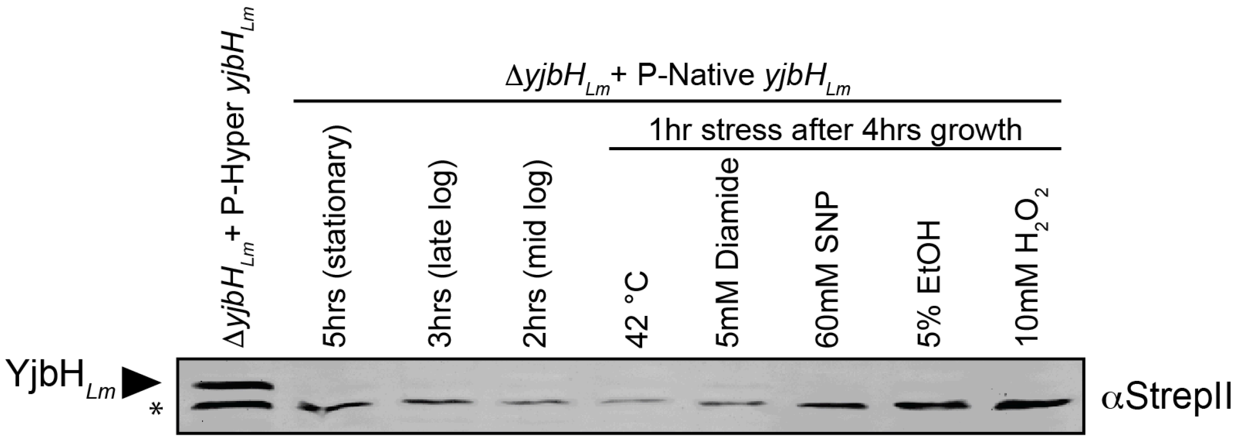

Figure S1. Natively expressed YjbH*Lm* is undetectable by immunoblot. YjbH was detectable when overexpressed from the P-Hyper promoter in the Δ*yjbHLm* background, shown here at early stationary phase (five hours of growth after subculture). However, YjbH*Lm* was undetectable when expressed from its native promoter in any growth phase tested and after treatment with heat, diamide, SNP, EtOH, or H_2O_2 stress. Cultures were exposed to the stated stressors for 30 minutes (not shown) or one hour in separate trials, with no effect seen on YjbH. This image is representative of three independent experiments. * denotes nonspecific band, which indicates approximately equal loading.

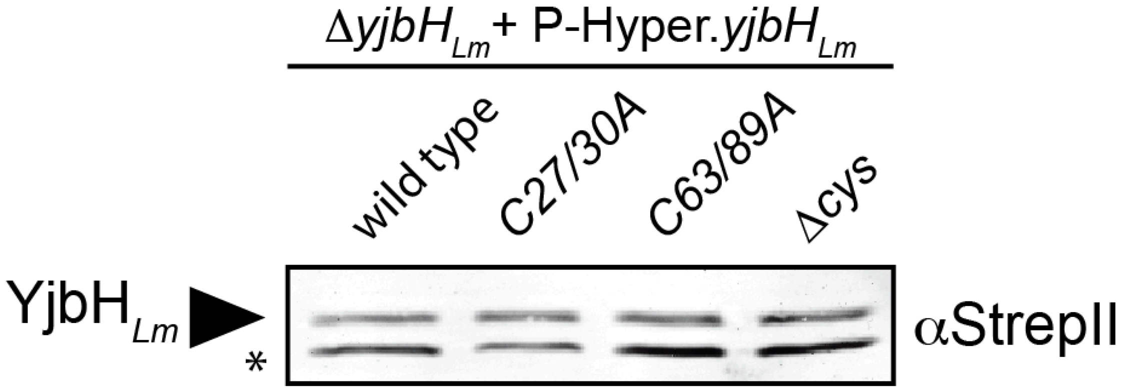

Figure S2. YjbH$_{Lm}$ cysteine mutants are equally as stable as wild type YjbH$_{Lm}$. YjbH, YjbH$^{C27/30A}$, YjbH$^{C63/89A}$, and YjbH$^{\Delta cys}$ are all overexpressed in a Δ*yjbH*$_{Lm}$ background. This is a representative image of three independent experiments. All single cysteine mutants phenocopied their corresponding double mutant in plaque assays, SNP sensitivity, and LLO secretion, and therefore only the double mutants and the quadruple mutant were tested for stability here. * denotes nonspecific band, which indicates approximately equal loading.

Table S1. *Listeria monocytogenes* strains used in this study.

Strain	Description	Reference or Source
10403S	Wild type	(1)
MLR-L081	Δ*yjbH*	(2)
MLR-L273	*P-spxA1::Tn*	(2)
MLR-L354	Δ*yjbH P-spxA1::Tn*	This study
MLR-L905	Δ*yjbH* pPL2.P-native.*yjbH*	This study
MLR-L906	Δ*yjbH* pPL2.P-native.*yjbH*^{C27A}	This study
MLR-L907	Δ*yjbH* pPL2.P-native.*yjbH*^{C30A}	This study
MLR-L908	Δ*yjbH* pPL2.P-native.*yjbH*^{C63A}	This study
MLR-L909	Δ*yjbH* pPL2.P-native.*yjbH*^{C89A}	This study
MLR-L910	Δ*yjbH* pPL2.P-native.*yjbH*$^{C27/30A}$	This study
MLR-L911	Δ*yjbH* pPL2.P-native.*yjbH*$^{C63/89A}$	This study
MLR-L912	Δ*yjbH* pPL2.P-native.*yjbH*$^{\Delta cys}$	This study
MLR-L913	Δ*yjbH* pPL2.P-native.*yjbH*$_{Bs}$	This study
MLR-L526	Δ*yjbH* pPL2.P-Hyper.*yjbH*	This study
MLR-L914	Δ*yjbH* pPL2.P-Hyper.*yjbH*$^{C27/30A}$	This study
MLR-L915	Δ*yjbH* pPL2.P-Hyper.*yjbH*$^{C63/89A}$	This study
MLR-L916	Δ*yjbH* pPL2.P-Hyper.*yjbH*$^{\Delta cys}$	This study

Table S2. Whole-cell proteomics revealed proteins less abundant in Δ*yjbH* than wild type.

Gene Locus	Predicted Function	Protein	Wild Type[a]	Δ*yjbH*$_{Lm}$[a]	*p*-value
lmo2102	Converts glutamine to glutamate and ammonia	PdxT	16.0	n.d.	0.001
lmo0202	Listeriolysin O	LLO	15.0	0.4	0.001
lmo0399	PTS fructose transporter subunit IIB	-	11.0	1.0	<.001
lmo2006	Acetolactate synthase	AlsS	22.0	2.7	0.004
lmo0355	Fumarate reductase subunit A; succinate dehydrogenase activity	-	11.0	1.7	0.001
lmo1530	Queuine tRNA-ribosyltransferase	Tgt	6.0	0.8	0.013
lmo0415	Peptidoglycan deacetylase	PdgA	19.0	4.0	0.004
lmo0401	Alpha-mannosidase	-	5.0	n.d.	<.001
lmo0536	6-phospho-beta-glucosidase	-	4.0	n.d.	0.025
lmo2101	Pyridoxal 5'-phosphate synthase subunit	PdxS	145.0	33.9	0.001
lmo0398	PTS sugar transport subunit IIA	-	4.0	n.d.	<.001
lmo2079	Hypothetical lipoprotein	-	4.0	0.4	0.011
lmo2569	Peptide ABC transporter substrate-binding protein	-	4.0	0.4	0.005
lmo1737	Oxidoreductase	-	4.0	0.4	0.016
lmo0181	Sugar ABC transporter substrate-binding protein	-	20.0	5.3	0.002
lmo2487	Hypothetical protein	-	11.0	3.4	0.009
lmo2767	Hypothetical protein	-	3.0	n.d.	0.007
lmo0786	FMN-dependent NADH-azoreductase 2	AzoR2	6.0	1.6	0.044
lmo1676	Menaquinone-specific isochorismate synthase	MenF	14.0	5.2	0.038
lmo2802	rRNA small subunit methyltransferase G	RsmG	3.0	0.3	0.017
lmo0487	Putative hydrolase	-	3.0	n.d.	0.006
lmo0814	Nitronate monooxygenase	-	3.0	n.d.	0.039
lmo1592	Probable tRNA sulfurtransferase	ThiL	4.0	1.0	0.025

lmo1604	2-cysteine peroxiredoxin	-	7.0	2.3	0.031
lmo1221	Phenylalanine-tRNA ligase alpha subunit	PheS	6.0	2.4	0.012
lmo0689	Chemotaxis and phosphorelay signal transduction	CheV	2.0	n.d.	0.001
lmo1936	Glycerol-3-phosphate dehydrogenase	GpsA	15.0	6.1	0.018
lmo0856	UDP-N-acetylmuramoyl-tripeptide-D-alanyl-D-alanine ligase	MurF	5.0	1.9	0.023
lmo2779	Ribosome-binding ATPase	YchF	10.0	4.4	0.005
lmo1454	RNA polymerase sigma factor	SigA	7.0	3.1	0.032
lmo1401	Hypothetical protein	-	7.0	3.3	0.023
lmo1902	3-methyl-2-oxobutanoate hydroxymethyltransferase	PanB	5.0	2.2	0.032
lmo1937	GTPase	Der	9.0	3.9	0.024
lmo1892	Penicillin-binding protein 2A; glycosyltransferase	PbpA	2.0	0.3	0.015
lmo2125	Maltodextrin-binding protein	-	23.0	11.0	<.001
lmo1710	Flavodoxin-like domain	-	8.0	3.8	0.044
lmo2556	Fructose-bisphosphate aldolase activity	FbaA	64.0	31.5	0.003

[a]Average spectral peptide counts from three independent samples are listed. n.d. indicates no peptides were detected.

Table S3. *E. coli* strains used in this study.

Strain	Description	Reference or Source
XL1 Blue	For vector construction	Stratagene
SM10	For transconjugation	(3)
BTH101	For BACTH system	(4)
DB3.1	For vector construction	Invitrogen
MLR-E006	pPL2	(5)
MLR-E234	pPL2t	(6)
MLR-E531	DB3.1/pUT18x	This study
MLR-E532	DB3.1/pUT18Cx	This study
MLR-E533	DB3.1/pKNT25x	This study
MLR-E534	DB3.1/pKT25x	This study
MLR-E080	SM10/pPL2.P-native.*yjbH*	This study
BRR-092	SM10/pPL2.P-native.*yjbH*^{C27A}	This study
MLR-E150	SM10/pPL2.P-native.*yjbH*^{C30A}	This study
BRR-106	SM10/pPL2.P-native.*yjbH*^{C63A}	This study
BRR-107	SM10/pPL2.P-native.*yjbH*^{C89A}	This study
BRR-120	SM10/pPL2.P-native.*yjbH*$^{C27/30A}$	This study
BRR-238	SM10/pPL2.P-native.*yjbH*$^{C63/89A}$	This study
BRR-113	SM10/pPL2.P-native.*yjbH*$^{\Delta cys}$	This study
MLR-E522	SM10/pPL2t.P-hyper.*yjbH*	This study
BRR-143	BTH101/pUT18Cx.*zip*; pKT25x.*zip*	This study
BRR-291	BTH101/pUT18x.*yjbH*; pKNT25x	This study
BRR-144	BTH101/pUT18x.*yjbH*; pKNT25x.*spxA1*	This study
BRR-169	BTH101/pUT18x.*yjbH*; pKNT25x.*spx*$_{Bs}$	This study
BRR-204	BTH101/pUT18x.*yjbH*$^{C27/30A}$; pKNT25x.*spxA1*	This study
BRR-227	BTH101/pUT18x.*yjbH*$^{C63/89A}$; pKNT25x.*spxA1*	This study
BRR-191	BTH101/pUT18x.*yjbH*; pKNT25x.*lmo0218*	This study

BRR-247	BTH101/pUT18x.*yjbH*; pKT25x.*lmo0256*	This study
BRR-248	BTH101/pUT18x.*yjbH*; pKT25x.*lmo1258*	This study
BRR-249	BTH101/pUT18x.*yjbH*; pKT25x.*lmo1387*	This study
BRR-250	BTH101/pUT18x.*yjbH*; pKT25x.*lmo1636*	This study
BRR-251	BTH101/yjpUT18x.*yjbH*; pKT25x.*lmo1647*	This study
BRR-206	BTH101/pUT18x.*yjbH*; pKNT25x.*lmo1782*	This study
BRR-252	BTH101/pUT18x.*yjbH*; pKT25x.*lmo2390*	This study
BRR-228	BTH101/pUT18x.*yjbH*$^{C27/30A}$; pKNT25x.*lmo0218*	This study
BRR-253	BTH101/pUT18x.*yjbH*$^{C27/30A}$; pKT25x.*lmo0256*	This study
BRR-254	BTH101/pUT18x.*yjbH*$^{C27/30A}$; pKT25x.*lmo1258*	This study
BRR-255	BTH101/pUT18x.*yjbH*$^{C27/30A}$; pKT25x.*lmo1387*	This study
BRR-256	BTH101/pUT18x.*yjbH*$^{C27/30A}$; pKT25x.*lmo1636*	This study
BRR-257	BTH101/pUT18x.*yjbH*$^{C27/30A}$; pKT25x.*lmo1647*	This study
BRR-208	BTH101/pUT18x.*yjbH*$^{C27/30A}$; pKNT25x.*lmo1782*	This study
BRR-258	BTH101/pUT18x.*yjbH*$^{C27/30A}$; pKT25x.*lmo2390*	This study
BRR-259	BTH101/pUT18x.*yjbH*$^{C63/89A}$; pKNT25x.*lmo0218*	This study
BRR-260	BTH101/pUT18x.*yjbH*$^{C63/89A}$; pKT25x.*lmo0256*	This study
BRR-261	BTH101/pUT18x.*yjbH*$^{C63/89A}$; pKT25x.*lmo1258*	This study
BRR-262	BTH101/pUT18x.*yjbH*$^{C63/89A}$; pKT25x.*lmo1387*	This study
BRR-263	BTH101/pUT18x.*yjbH*$^{C63/89A}$; pKT25x.*lmo1636*	This study
BRR-264	BTH101/pUT18x.*yjbH*$^{C63/89A}$; pKT25x.*lmo1647*	This study
BRR-265	BTH101/pUT18x.*yjbH*$^{C63/89A}$; pKNT25x.*lmo1782*	This study
BRR-266	BTH101/pUT18x.*yjbH*$^{C63/89A}$; pKT25x.*lmo2390*	This study
BRR-153	BTH101/pUT18x.*yjbH;* pKNT25x.*clpX*	This study
BRR-270	BTH101/pUT18x.*yjbH;* pKT25x.*clpC*	This study
MRC-089	BTH101/pUT18x; pKT25x.*spxA1*	This study

METHODS

Immunoblotting for YjbH Protein. Overnight *L. monocytogenes* cultures were subcultured 1:10 into BHI media containing streptomycin and incubated for five hours at 37 °C, shaking. For cultures in Figure S1, the incubation period is as noted on the figure: either 2, 3, or 5 hours of growth. For the stressed cultures in Figure S1, the noted sublethal stressor was added to the culture for the last 30 minutes or one hour of a total of five hours growth at 37 °C before harvesting. These stressors included: stationary incubation at 42 °C heat; 5 mM diamide (Sigma-Aldrich); 5% ethanol (Fisher Scientific); 60 mM sodium nitroprusside (Sigma-Aldrich); and 10 mM hydrogen peroxide (Invitrogen). At the end of the stress period, OD_{600} measurements were taken with a spectrophotometer. Cultures were pelleted, washed, and resuspended in a volume of 0.1% NP-40 + 0.1 mM PMSF equal to 10X the OD_{600}. Zirconium beads (0.1 mm diameter, Benchmark Scientific) were added to each sample before bead-beating twice for 30 seconds each, keeping samples on ice in between. Samples were centrifuged at 4 °C for 15 minutes and then boiled in loading dye. Gel electrophoresis was used to separate each sample. Proteins were then transferred to PVDF membrane (Bio-Rad), and the membrane was blocked with Odyssey blocking buffer (LI-COR Biosciences). Proteins of interest were detected using polyclonal rabbit anti-StrepII antibody (VWR) at a dilution of 1:2,000. Goat anti-rabbit (Invitrogen) antibody was used to detect the primary antibody, at a dilution of 1:5,000. Immunoblots were imaged on an Azure Biosystems Sapphire Biomolecular Imager.

REFERENCES

1. **Bécavin C**, **Bouchier C**, **Lechat P**, **Archambaud C**, **Creno S**, **Gouin E**, **Wu Z**, **Kühbacher A**, **Brisse S**, **Pucciarelli MG**, **García-del Portillo F**, **Hain T**, **Portnoy DA**, **Chakraborty T**, **Lecuit M**, **Pizarro-Cerdá J**, **Moszer I**, **Bierne H**, **Cossart P**. 2014. Comparison of widely used *Listeria monocytogenes* strains EGD, 10403S, and EGD-e highlights genomic variations underlying differences in pathogenicity. mBio **5**:e00969–14.

2. **Reniere ML**, **Whiteley AT**, **Portnoy DA**. 2016. An *in vivo* selection identifies *Listeria monocytogenes* genes required to sense the intracellular environment and activate virulence factor expression. PLoS Pathog **12**:e1005741–27.

3. **Simon R**, **Priefer U**, **Pühler A**. 1983. A broad host range mobilization system for *in vivo* genetic engineering: Transposon mutagenesis in Gram negative bacteria. Nat Biotechnol **1**:784–791.

4. **Karimova G**, **Pidoux J**, **Ullmann A**, **Ladant D**. 1998. A bacterial two-hybrid system based on a reconstituted signal transduction pathway. Proc Natl Acad Sci USA **95**:5752–5756.

5. **Lauer P**, **Chow MYN**, **Loessner MJ**, **Portnoy DA**, **Calendar R**. 2002. Construction, characterization, and use of two *Listeria monocytogenes* site-specific phage integration vectors. J Bacteriol **184**:4177–4186.

6. **Whiteley AT**, **Pollock AJ**, **Portnoy DA**. 2015. The PAMP c-di-AMP is essential for *Listeria monocytogenes* growth in rich but not minimal media due to a toxic increase in (p)ppGpp. Cell Host and Microbe **17**:788–798.
